# Supplementary material for: Patterns of Intron Gain and Loss in Fungi
Source: PLoS Biol. 2004 Nov 30;2(12):e422. doi: 10.1371/journal.pbio.0020422 (PMC532390; doi:10.1371/journal.pbio.0020422)
Supplement: Table S1 — Also available at http://genes.mit.edu/NielsenEtAl/. (4.3 MB ZIP). [file pbio.0020422.st001.zip › NielsenEtAl/html/1391.html]

AN8671.1.NCU02036.1.MG02784.1.FG08650.1


```
 CLUSTAL W (1.82) Multiple Sequence Alignments - Introns Inserted


Sequence 1: NCU02036.1	2126 aa
Sequence 2: MG02784.1	2037 aa
Sequence 3: FG08650.1	2078 aa
Sequence 4: AN8671.1	2234 aa
Alignment Length: 2343 aa
Number Identitical Residues: 451 aa
Alignment Score (without introns) 25299


MG02784.1 	-----MDPNEVYEEIMASYT~QLEKFPEDMHLFCPKLSEDDEVDYEDPNEEVWDGEVQIT
NCU02036.1	MEYPELGTADVQMAMMEMHQ~AFEEFPPDAHPICPLLSDDDDTDYALP--DLPDANGTTV
FG08650.1 	--------MDLHEELNQWYE~KIQTFDGECHLLCPRINDEDNENYRIQ----HDPESAIS
AN8671.1  	------------MEFMKIVA2KLRALPDEVHILCPRQNDDDHARYDDE-------NELHE
          	              :       :. :  : * :**  .::*.  *          :    

MG02784.1 	AEQKKERIEDSDKRLHIATTASLLLGISKDAASAWLDGWIERAEKNLTTCPNCIRAWHKK
NCU02036.1	AEKQK-RIADGFERRRITYGLSLLLVMP--EAGKWINEWKERVNFFLTKCDQCARTWHRT
FG08650.1 	QEEKTKRIEQGRTRLEVTYWNCLIFGFDEDSQGKWGKTLGDRLNLCLKHCADCVYNWHMN
AN8671.1  	SGRKVAEMVAEAKERREEFLSCMRILAYNEEVEELQKWIWQKIDDTLEKCDLCIKQYYMG
          	  .:  .:     . .     .: :   ..      .   :: :  *  *  *   ::  

MG02784.1 	RKSFLKLLGD2KFG---DEVARIMAQKLDEFDKNRIDRGLLKAESLLK----EHGPMKSS
NCU02036.1	RQQFIRAIDQ2YTSRDNAELLRNLQKNLEAFDEERITRGLKRAKFILE----RDGPMPTT
FG08650.1 	RRPLLQQFQD~KWS---EEVANEIKAMLQSKDVIRIDQSLQWAKAAIEKIIATCSVFKKS
AN8671.1  	KIWLMEQLKE~NYD---DEDIEKFSRMLDECDIKRITRNLATATATLR-----ALPADQI
          	:  ::. : :   .    *  . :   *:  *  ** :.*  *   :.            

MG02784.1 	TLTKKEPTAVVALYEALCCMSYLSR-PQNRMHFNFVFSEIQKIKRPLRLGS-GVIPTMTF
NCU02036.1	KLVRIELWPCLALFESLCCMAYLNTNKANLEIFNYVFEHVQ-PKKPLKMVG-GAIPTMTY
FG08650.1 	QLGEHLSEVHISVYEALCCMPYMED-PERRTTFQFVFQRLQ-GKSGLKLGTKEPLPGMTY
AN8671.1  	GLHVLDRASLLSIFETLSCDAMLRNDSLLTEHFDEPFRLIQ-TKRTLKVSD--YIPAVTR
          	 *        ::::*:*.* . :  .      *:  *  :*  *  *::     :* :* 

MG02784.1 	FLFQEDEDGYRSGFAKAAWETMPPGSLTANQFEWAVQDYLASEIS--AVFNPSVPEAR--
NCU02036.1	FLFEEYP--VHRAFAEAQWQRMLPESLTDEEWEWAVRSGLKSHIL--GHRG-IQPPAR--
FG08650.1 	FIFDLRNQ-DRRTWATNNYQNLDSNAMTLDQFDWAVSGGLSRAIDDISRRDPLSPESWPD
AN8671.1  	FLFSTNHS--RSHWAVHSWMRYQR-APTAAEFDWAIKEGLLDALR----AASQQPVQIAA
          	*:*.   .  :  :*   :      : *  :::**:   *   :       .  *   . 

MG02784.1 	IKRFWDGIAIIIPSMSADFVLHTLRGMEVQP---DIYHIAFHHLSYVQSPEVFATLIKGI
NCU02036.1	LCRFWSGFLLILRAMSEKSILENLRGMEVQP---NVYFLALENLGTNNVP-ALSMILKAL
FG08650.1 	IEQFWQSFNEIMRTLNADVIPQRLRDLELKPGSLSIYDLLFRHIQDCPAESVLVTNIIVL
AN8671.1  	IQRLWRGMQFIAKRLDKEQITHNLRALEIDPCRLSVEHLAFQTAS-------LRWTLNTI
          	: ::* .:  *   :. . : . ** :*:.*   .:  : :.          :   :  :

MG02784.1 	ISLIQKSPKAFWDALADVSPAIFTQHTLGSKAFPAMMARSLEPNMLVRVGDKD---IFLL
NCU02036.1	RTLMERSSKAFWDAYDQVTPSMIVEEIFKSPAFRPFLNQSLEPDMMVEDQENNGQLIPAL
FG08650.1 	TNFFKKAPRAFWDVIGDARPTVIPDLVLKSRVYKNLLRQSLDNCWTGFDSGASR--APFP
AN8671.1  	QIFLEKTPGDFWDAMQTISPQAIVEVVFYNPQLESFLMEATEGQAYEKLAMKD------M
          	  :::::.  ***.     *  : :  : .     :: .: :          .       

MG02784.1 	SALVKILMESLQPTGRTDLCDSTLHSLMDRFRSEP--SREGKSACAHAALMSMICTLE-C
NCU02036.1	AEWVRAFIRSLPSARRSDVCETLLRHLFETLRVDAGAAPEAKATCILAGLVTLRECLDGY
FG08650.1 	TSWVNPWLESIQRDQRYDACDGFMHTLLETLAKDQTIGEPGRAACARAGFDALQLTMSTF
AN8671.1  	LSWVNPFMSSLKGPHQPTACRSLVYQLIDRLQDTR-FPNLARYHCFEVGLVSLLHTLRSF
          	   *.  : *:    :   *   :  *:: :         .:  *  ..: ::   :   

MG02784.1 	LSDP-KLSFSTALVYIDITYNIVLRHKDFVINCASLKNGDQ---YNVGLSEAALKVIDLA
NCU02036.1	LSLP-SFDTGTSLIMVNQLLNRVIQYKEVIIDATRLKAADK---YNVGLSQAAIAIIQSA
FG08650.1 	VDQETKIGAGTTHLYACCAFNLPMKYKDLILRNLRAPLQEREGWQTFQVANAARLAMQTA
AN8671.1  	TDHE-SSRESVARVVLSETLGVVSEYINTILKPPQFNVEQG---RQRAIKSLCMDVIRNT
          	 .   .   ..: :      .   .: : ::        :        : . .   :  :

MG02784.1 	LTLDSETFERESLAIKEAKKLSYAVERRSSKLWDAFIDHLEPSK-TTSLANVILGATLGL
NCU02036.1	LALDAKATLMEWDAFVKEKPVQDAVNRDSGALWESFLELVWNG--QTDLAKAMLRATMPL
FG08650.1 	MKLDMKIFAEEYSACLDGNQLQSTVIRDSKVFWQGVVEMFDVSSERIDLAKDIILSLEPL
AN8671.1  	LALECQSLKSDYEVILKHGSLQHGVSTYSSSIWDAVIMHLHEN--DLALSTSALLGILPL
          	: *: :    :  .  .   :.  *   *  :*:..:  .  .     *:.  : .   *

MG02784.1 	RGVEKFLPRKREAVVKDPQKLNFNQKFEATAESIGRLIGRVSEFSSQELRMLSEDHR--A
NCU02036.1	RSIERFIPKRKDQLKN--EHAKFNRRFQQQTAAIGKMLGRLSDFNPADLDSFCSDPQSNT
FG08650.1 	VGVEQVRALKGNDQLTD-SSKSFNKSLGETVDILGNLLGRVSELDATDLNTLFNDRL--A
AN8671.1  	VGLEKFSLKEG----TKPEKTHFNTIYGHLTHISCQIIERLADFKPEHLDELFKSQD--T
          	 .:*:.   .     ....   **      .    .:: *::::.. .*  : ..    :

MG02784.1 	LGAIVACLTHGEENISDAAMNFIKMYTGEDSRSDALEKMLSSHPELFLRSFSDAVGKKIP
NCU02036.1	IQPIVATLIHGEEAIREAGFELIKAITGEMTPSEAVNKMLEEYFSQFLEAFTGAVNHITN
FG08650.1 	FQGTIALSTHGESALAEAASELLKSWTGELSQSGAFEQMSQLHPDQTLSSIVWALERVLK
AN8671.1  	GSALVSTLFAADHNTYQAAVDLIKNVSGQSARRDAISHLLQSFFTTTLYGLSWSFRRISN
          	    ::    .:    :*. :::*  :*: :   *..:: . .    * .:  :. :   

MG02784.1 	HPANAEMFPPVAKVLGWYRDILDAVTDSASGLLRR-DLSEEVAEA-VKYWWKRQWVFLGY
NCU02036.1	AKDSNNPWSHMQHLLKCCEFVLNSLSDPSVGQLRRKTLTPAEHAI-VKGWWDCEWRAVGH
FG08650.1 	PP---YPWGPIRPILNMSRDILRGLTDPISGVLRVKTLEPMSAAK-VLRWWNEQWRFVST
AN8671.1  	MK----TFASAPRMIHTGTDIVDILCDTQTGILRTRKLVDRKETLSLQKLWEYLWQALTT
          	       :     ::     ::  : *.  * **   *       ::   *.  *  :  

MG02784.1 	FFNQTRHWSEQYVPKETMETLCREAVEHADRLLAEDGLVANAIKCLVKSDGQNGDARTAA
NCU02036.1	SFGSMRLWHVK-VDKKVMEDFCRDVMELAEKLLAQDGLMASALSSPQSISATNGDDGSEG
FG08650.1 	ACRNIDGWSRY-IQNAIMTEFCREIMELAEALVAEDGLITSAIS------KALGKSEEDT
AN8671.1  	IFYETEEWHRRGNDKAVMLEFCRDSIQFADLLFSQYGVFLSAVID------ADPTQEATA
          	   .   *      :  *  :**: :: *: *.:: *:. .*:                 

MG02784.1 	MKVVLDQPRVITPAYFAMLQLRDQWLLGVFARSVGKLVTRLNEFDMALPPRPLQMLRDIC
NCU02036.1	MKLVLEPPRIHSLPLVDMLQLRDKYLIAGIIEITKKLIKRLQDNGMDLPPKTVAYLNNML
FG08650.1 	MTSILGAPKQYFSGMEIMIRLKDKWLVDVTVRVICKILTRLRENKLEIAPSSRKLITDAC
AN8671.1  	RENFLKPPTATMSGMAKWLRLKDEYLATTLVGLVSKILRRLGELNVTTLKEDALSFIEGV
          	   .*  *          ::*:*::*         *:: ** :  :         : :  

MG02784.1 	IKT--HDGR--FKVNSNLRPAQKAELLRALG--TDSSDDEEVQFVSEAQKPPKAG-MPKK
NCU02036.1	KKRKMTDGRTDYPIKTNLTNEQRIELLKALG--DDAA--IEAQFVGGKPVKQEVK-PVMK
FG08650.1 	LPTN-IPGK--YVRSTNMTDQQRAELLQALGQSDDEVQIIQLGFAGKGTTSSKSKDPVKK
AN8671.1  	AINN--------TVKTIITPREKAELVRALEAYYKKPVVTASTASLKKQSMITAFAKPAE
          	   .          .: :   :: **::**    .                        :

MG02784.1 	QSSLAAWSKSGFSGETGTAPTAPARTTS------KATSDEQQLSIMQSIKAQVQSERKPK
NCU02036.1	QAKLD-FSKASLKDHIASLTPAFEKATE------RRKN-----ALLETMKAEAPAKAPPK
FG08650.1 	QSKIDAWSKSGTS-SSGTITTPSSRSNRDDVHELSRSIDSPILKQLDAQKAREQARAKSK
AN8671.1  	SSASPSPSRATDEFNESDVSDDVLLQLSR----SVELSKERFATAEAKRRAEKPVRPLSV
          	.:   : *::  .   .  .                  ..         :*.   .  . 

MG02784.1 	PQMLSAAAIKS----NQSSLKEARAKEQAERAKANAASVARAKAIREAFSSGNKIVAGEG
NCU02036.1	IDSKAADKIK-----------EARRLEREAKAKRDAEAIARAKALRAPP----KLVQGEG
FG08650.1 	TNAMLKSKSKVPDQKSISALKESRQKEKLEKQKRDAAAIAKAKALRGET------VPGEG
AN8671.1  	TPKPVATPAPMKSSLAIQSFREKREKEREAKKKRDLAELARLKRSAPTG--VAEQTAMQG
          	            ..   .:  * *  *:  : * :   :*: *          . .  :*

MG02784.1 	SGLQGISGVLGKDHAPT-KSEIMVDSSEEE------GGDSDD-EMSDLFGAGQAGKDAAA
NCU02036.1	SGLAGVAGVRGKDHAPVLKDEIMVGSSSED------EDESDDDELITLHAAGKKNMTEAE
FG08650.1 	SGLLGLG-LSGKDHGKS---EIMVNSSDEESDEDEEEDEDADNELAALSTGGLKTMSEAE
AN8671.1  	SAITGLG-VKGKDHSAPADSMMVSSGSETES-----ESEDELDRELFGPKASSKPDAVKA
          	*.: *:. : ****.   .. :: ..*. ::      .:.  ..      ..        

MG02784.1 	QRLKQLAAKQMV-PVKKTKIQRSKKDLRARITPPMERLHEAILEWDIFHEGSDPPNG--Y
NCU02036.1	RRAHRLLAEKMRGPVKKVKIERSAKDMRARLIPPMDVLHQAILEWDIFHEGNDPPNG--Y
FG08650.1 	KRLSKAMQDMVRRPVKKVRQQRSVKEMRARLIPPMDRLHNTILSWDIFHGGNDPPKG--P
AN8671.1  	YEESKKKSLKIQGPVKKIKQVRSAKDMRARLAPDLSSLHRTLLSWDFFATGDLPPSSGRT
          	 .  :     :  **** :  ** *::***: * :. **.::*.**:*  *. **...  

MG02784.1 	KCKEVAKSYTNPVDYRETFFPMLVNEAWRSFVTSKDESTAKPFDIKITTRTSVDKFLEVA
NCU02036.1	RCDHVSDTYQDPVSYKQTFFPLLINEAWRSFVTAKDETTSKPFGIKVLSRMTVDKFMEVT
FG08650.1 	VASEVATKYSDPKTYQDTFFPLLASEAWRSFVTAKDEITSQAFGMKIASRASVDSYLEAT
AN8671.1  	DYSLVSNSFRDPIEYQKTFEPLLILEAWQGFQASKEEGGFKTFEIKVATRLSVDAFVEVN
          	  . *: .: :*  *:.** *:*  ***:.* ::*:*   :.* :*: :* :** ::*. 

MG02784.1 	TSMPISANKDKDRDRLSDGDIVLFSTAQDPLSSKESPHCLARVFKTGRKRGVLEVTYRIT
NCU02036.1	ASVPVAINKDR---QLSEGDIVIISKGQNPLQEPEELHCLSRIWKTTYKKDNVEVVYRLN
FG08650.1 	FTMPVVQNRER---GVSEGDILLVSESENPLGDQTARHCLARVHRITYKKELVEITYRVA
AN8671.1  	TNMIGMDVKDT---GVFEADLVLLSKASNPATNPSVPHCLARVANISKKKGVVEVSYRVN
          	 .:     ::     : :.*:::.* ..:*  .    ***:*: .   *:  :*: **: 

MG02784.1 	SKGNSRFASDHLLPGNDLRGLRITNMTTVEREFAALESLQYYDLMTEVLDAEPSPILNFS
NCU02036.1	AKGNQILP--ALLPGSEFTVVKITNMTTIEREYAALESLQYYDLMDEVLRAEPSPMLSFG
FG08650.1 	SRNNPMTQ--VLTPNVSVFGVKITNMTTIEREYAALESLQYYDLMDEILNAKPSPILRYD
AN8671.1  	PGNPFINS---LGPGATIYGAKITSLTPLEREFGALMALQYYDLCEEIVRARPSPILNYS
          	. .        * *.  .   :**.:*.:***:.** :******  *:: *.***:* :.

MG02784.1 	NDRIQSYQDNYQLNRGQAAAIINAKENDGFTLVQG~PPGTGKTKTIIAMVGALLTGKISR
NCU02036.1	EENIKNTMKNWNLNPGQAKAILNAKENDGFTLIQG~PPGTGKTKTIVAMVGCLLTGVLKN
FG08650.1 	EAKVNNCMQNYSLNHGQAMAVLGAHDNDGFTLIQG2PPGTGKTKTIVAMVGTLLSEQLSQ
AN8671.1  	DASLKPLVDNYNVNPAQAKAIKSAMDNDAFTLIQG~PPGSGKTKTIVALVGALLTNALKD
          	:  ::   .*:.:* .** *: .* :**.***:** ***:******:*:** **:  :. 

MG02784.1 	APP---------TRIKPANGADEPMAQKLLVCAPSNAAVDELVLRLKAGIKDTNGNTHKI
NCU02036.1	PTAG-----VAIGRPGLGAAKNNAPAKKLLVCAPSNAAVDELVLRLKNGVKTQNGTTHQI
FG08650.1 	ASNQGFPVGVPLRPTGLQASNSQKRSKKLLVCAPSNAAVDELVLRLKAGVKTISGKTKSI
AN8671.1  	QGIVITR---PTAAINARPGARTTTSKKLLVCAPSNAAVDELVMRFKEGVKTIHGRHEKL
          	          .        .     ::****************:*:* *:*   *  ..:

MG02784.1 	NVLRLGRSDAINAAVRDVTLDELVKEKMDAALNVNGSGNSGPTDREKLHQEAGEIKVRVA
NCU02036.1	EVVRLGRSDAINSAVKDVTLDELVKAKLEAQLNKD----EGPTDREKLHQEAGEIKQKIA
FG08650.1 	NVLRLGRSDAINAAVRDVTLDELVKARLEGDQTKD----KAKANRDKLHEDAGKIKEQLA
AN8671.1  	SVIRLGRSDAINTNVLDVTLDELVNARLKQTSGEK----SGETDLQKIYVEHKAADTAFK
          	.*:*********: * ********: ::.     .    .. :: :*:: :    .  . 

MG02784.1 	ALREALEQARAAGDHGQTNSLQRNLDELRKKQGQIGAQIDRDKASGNTYAREAEIKRRNI
NCU02036.1	ELRPQLEAARTIDDRQLVNKYQREFDELKRRQAHIGARIDADKASGNTFARETEIKRRQV
FG08650.1 	ILRPLMEASKDHEDRTTYTKHSREFDALKRRQMDIGKQIDADKSSGNSVAREMEVRRRQV
AN8671.1  	EVRSKMDQCRAQG-LPVPEELQREFDLLKKKKTQLSHQIDNARDKNHSAARDADLNRRRI
          	 :*  :: .:         . .*::* *:::: .:. :**  : ..:: **: ::.**.:

MG02784.1 	QQSILSEAHVLCATLSGAGHDMFKSLQVEFETVIIDEAAQCVELSALIPLKYGASKCILV
NCU02036.1	QQEILDKAQVLCATLSGSGHEMFKNLNVEFETVIIDEAAQCVELSALIPLKYGCSKCILV
FG08650.1 	QQEILNNAQVLCATLSGSGHEMFRNLDVEFETVIIDEAAQCVELSALIPLKYGCYRCVLV
AN8671.1  	QQEIIDGAHVICATLSGSGHEMFQNLSIEFETVVIDEAAQSIELSALIPLKYGCSKCILV
          	**.*:. *:*:******:**:**:.*.:*****:******.:***********. :*:**

MG02784.1 	GDPKQLPPTVLSQSAARYGYDQSLFVRMQQNHPGKVHLLDCQYRMHPEISLYPSKEFYEG
NCU02036.1	GDPKQLPPTVLSQSAARYGYDQSLFVRMQKNHEKDVHLLDTQYRMHPEISSFPRAAFYEG
FG08650.1 	GDPKQLPPTVLSQSAAKFGYDQSLFVRMQQNHPDWVHLLDMQYRMHPEISMFPSREFYEG
AN8671.1  	GDPKQLPPTVLSKVASKYQYEQSLFVRMQSNHPNDVHLLDIQYRMHPAISMFPRTAFYDG
          	************: *::: *:********.**   ***** ****** ** :*   **:*

MG02784.1 	LLADGSDMAKLRQQPWHDNPLLGPYRFFDVEGIQERGSRGQSLVNTNEINVAIQIYTKFQ
NCU02036.1	LLQDGDDMAKSRLQPWHRSTLLGPYRFFDVRGSQERGPKNQSLVNEEELKVAMQLYRRFK
FG08650.1 	QLADGQNMHELRQQPWHQSALLGPYRFFDVQGVQARGHKGQSLVNTRELDVAMQMYDRFS
AN8671.1  	RLQDGPDMARLRTRPWHQSELLSPYRFFDVQGLHQSAPKGHSLVNFAELKVAMQLYERLL
          	 * ** :* . * :*** . **.*******.* :  . :.:****  *:.**:*:* :: 

MG02784.1 	ADYASSIDMKGKIGIITPYKAQLFALRQKFQERWGEGVLEDIEFNTTDAFQGRECEIIIF
NCU02036.1	ADYG-NVDLKGKIGIITPYKAQLYRLRSQFAQRFGDAITDEIEFNTTDAFQGRECEIIIF
FG08650.1 	NEYG-DCDLTGKIGIITPYKAQLFELRNRFRSRYGEGITDIIEFNTTDAFQGRECEIIIF
AN8671.1  	KDFK-EYDFSGKVGIITPYKGQLRELKTQFANKYGNAIFNMIDFNTTDAFQGREAEVIIF
          	 ::  . *:.**:*******.**  *: :* .::*:.: : *:***********.*:***

MG02784.1 	SCVRASPTGGIGFMTDIRRMNVGLTRARSSLWILGDSRALRQGEFWNKLIEDSKARDRYT
NCU02036.1	SCVRASPTGGIGFMTDIRRMNVGLTRARSSLWILGDSRALMQGEFWAKLIEDSKRRDRYT
FG08650.1 	SCVRASSTGGIGFMTDIRRMNVGLTRAKSSLWILGDSRALVQGEFWKKLIEDAQSRDRYT
AN8671.1  	SCVRASNKG-IGFLSDIRRMNVGLTRAKSSLWVLGNSQSLVQGEFWRSLVTDARQRNVYT
          	****** .* ***::************:****:**:*::* ***** .*: *:: *: **

MG02784.1 	QGSIISQLRN--SKAIKGSYVPPPPAVPAPIGNVQRRGTEGSDVEMFDVND--------G
NCU02036.1	TGNIMGMLSQ--AGPQLSAAAFEALAITS-TGNTRESTPSGIKREVVEVKDEDSDVQMGD
FG08650.1 	KGDILSMFRKPLEKAKPGAYLPPPSKIQDREVVIRDTSYTMSSVPSSRSNS--------P
AN8671.1  	DGDILKLLEKPQFTGYKNVEMAEADASDSPAPSRPLSASSVSRPPSVSLENLPPESPVSA
          	 *.*:  : :.      .     .                         :.  ..   . 

MG02784.1 	RARQQSLPQQAPPQN---------------------------------------------
NCU02036.1	RSSFRGSSPPAAAVG---------------------------------------------
FG08650.1 	TSIAAQKPTPVSQIP---------------------------------------------
AN8671.1  	GASGKDTPPLTKPEGPSGGGNGLDETRNCGICGSFAHMTPNCDNEESKEIARGNCFRCGE
          	 :     .  .    .:..... ..: ... ..: :  :.......:.. : ...  ...

MG02784.1 	--------------------------------------NMASHSSSAAPPIKIT------
NCU02036.1	--------------------------------------GSSAMGSSRPPSGRNTPSHTGN
FG08650.1 	--------------------------------------GIGASDETGIPP----------
AN8671.1  	VGHTKFNCTAERCTECGQFGHTSQTCRSTKLLSKQERMKISREELQHSRNQKQRAEKQRL
          	 . :. ..::. .:.... . ::.:. ::.  :...    .             ..    

MG02784.1 	------VPGQPPARAVSQAPPIRSSVDMG---RKRPASEEARDQSSAN~KR0SVSNHPHP
NCU02036.1	GFGSSPAPQQPQPTQYREGLPVIQETTFTPRVKKRPYEGGGGDQPGKK0QH~IDPIASRA
FG08650.1 	------RFAGPPVIHTSSSKPAG-------ELKKRPHEGQEINQP-VS~KR0IASDSKVR
AN8671.1  	RQLGGHDPKVPVISVSMDKPKTQGSDAPSKRKRTDSPSSNASKAPRTS~KG~NQGAPPNA
          	   ..     *      .      .       :. . .    . .  . :          

MG02784.1 	PTGPRAG-------APRPPP-~RPTDPS-----~-AMQALG~LAPPDRP-----------
NCU02036.1	PKGPRGYNPNTNNLPPRPPPQ~APSDPS-----~-AMEVLG~LAPPTRAPAARQQQQQQP
FG08650.1 	GGGLMGKFGQK---PPRPPK-~TPVDPS-----~-ALSVMG~LVPPERP-----------
AN8671.1  	PSGPKTQRRRVDADVPPPSRE0NLMKPSRDGPA2QAQVLRA2LLLVHRNNGQETEFDRSS
          	  *        .   * *. .    .** ...: .*    . *    *  .   . . ..

MG02784.1 	----AA0QQPP----------~------------PPQAP---------------------
NCU02036.1	QYDYQP~QQPPQYQPRPQQGA~PPPYPGHQQQRQPPAGPSRPGPPTGPSRLGGSGTGYQQ
FG08650.1 	----PP~SMPT----------~------------SPAIPAGPKIPTGPSNQGHR----KS
AN8671.1  	PCGRRK~TLTPSFGQSVDKHY1RVWPKSCGSAAATALRPTTDFKVAERSLTGHWSKERTT
          	  .      ...     ..        .  .   ..  *:     :  :  .  ..    

MG02784.1 	--------------RPPKQWKKKAPANVFLQPKR--------------------------
NCU02036.1	QQQQQPQQQQQQQQRPPPIVKKKAPVDPFIKRKKR-------------------------
FG08650.1 	LDGNNQTQQARRPPGPPPPLRKKAKPSLFVPKKR--------------------------
AN8671.1  	CKFRTGSTALSPLVNSPLGEARDSEEVQETSGARRSFDAYSLYRLVSQEPYGTPGLAAEE
          	 .             .*    :.:         : : .: :     :... .:.. ::..

MG02784.1 	-------------
NCU02036.1	-------------
FG08650.1 	-------------
AN8671.1  	CSERLVYQRQLSR
          	.:.    . . :
```
